# Working Together: Research- and Science-Based Regulation of BPA

**DOI:** 10.1289/ehp.1306963

**Published:** 2013-07-01

**Authors:** Linda S. Birnbaum, Jason Aungst, Thaddeus T. Schug, Jesse L. Goodman

**Affiliations:** 1 Director, NIEHS and NTP, National Institutes of Health, Department of Health and Human Services, Research Triangle Park, North Carolina, E-mail: birnbaumls@niehs.nih.gov; 2 Center for Food Safety and Applied Nutrition, Food and Drug Administration, College Park, Maryland; 3 Cellular, Organ and Systems Pathobiology Branch, Division of Extramural Research and Training, NIEHS, National Institutes of Health, Department of Health and Human Services, Research Triangle Park, North Carolina; 4 Office of Chief Scientist, Food and Drug Administration, Silver Spring, Maryland

Both the National Institute of Environmental Health Sciences (NIEHS) and the U.S. Food and Drug Administration (FDA) work to promote and protect public health. The NIEHS achieves this mission by conducting research, including toxicological studies, on agents of public health concern through its intramural laboratories, the National Toxicology Program (NTP), grants and contracts to research labs across the country, and interagency agreements. The FDA, in turn, reviews and uses information from these and other studies and, where needed, performs studies of its own to develop standards to ensure that the products it regulates meet its requirements, maximizing product benefits while protecting the public from unacceptable risks.

The FDA and NIEHS have a long history of working together to generate and evaluate toxicological data necessary both for setting standards and for informing important regulatory decisions. For example, collaborative research projects between the NTP (itself a collaboration involving the National Institutes of Health, FDA, and Centers for Disease Control and Prevention) and the FDA National Center for Toxicological Research (NCTR) have contributed to safety determinations and regulation of a number of ingredients in dietary supplements, animal feed, and cosmetics and have been important for assessing potential risks of both human and veterinary pharmaceuticals (NTP 2013).

For several years, the NIEHS and FDA have been working collaboratively to address potential health concerns about bisphenol A (BPA), a chemical used in manufacturing the packaging of some foods and beverages, in some medical devices, and in some thermal papers. In September 2008, the NTP completed a review of available research on BPA and concluded that there was “some concern for effects on the brain, behavior, and prostate gland in fetuses, infants, and children at current human exposures.” The NTP report also recognized the existence of substantial uncertainties, stating that “Overall, the current literature cannot yet be fully interpreted for biological or experimental consistency or for relevance to human health” [NTP-Center for the Evaluation of Risks to Human Reproduction (CERHR) 2008].

In 2008, the FDA, which has regulatory authority over many consumer and medical products containing BPA, issued a draft assessment of BPA (FDA 2008). In 2009, the FDA provided additional updates to the assessment and expressed its agreement with the the NTP’s perspective (FDA 2013). The FDA, like the NTP, also identified substantial uncertainties both in reported BPA research findings and in their implications for human health. Both agencies called for further research focused on key questions about BPA to help address and reduce these uncertainties.

Since that time, the FDA and NIEHS have worked both independently and collaboratively to address areas of concern and to reduce uncertainties, including collaboration to enhance the relevance and usefulness of planned research in assessing potential risks to human health. These collaborative efforts have resulted in a number of important advances in our understanding of BPA. Both the focus and careful planning of the research, as well as the research findings themselves, are highly relevant for approaching other similar issues. Important accomplishments include technological and methodological advances in critical assays [e.g., determining levels of the active form of BPA in blood (Patterson et al. 2013)] and assuring strong study design, performance, and analysis, including carefully addressing considerations such as control groups and statistical analysis (Delclos 2013).

The FDA/NCTR and the NIEHS Clinical Research Unit have incorporated these advances and perspectives in planning and carrying out a comprehensive investigation of BPA pharmacokinetics in human volunteers. The FDA/NIEHS collaboration has also more generally supported approaches to the design and conduct of studies that enhance study utility and reliability for supporting sound decision making. We have worked together to support both large-scale regulatory studies that examine multiple biological end points of concern, and new and unprecedented collaborations between academic and regulatory scientists that enable smaller-scale exploratory studies of mechanistic and other end points not traditionally evaluated in regulatory toxicological studies.

The results of our collaborations to date have been especially important in improving the understanding of how BPA is metabolized and handled once in the body. This has greatly reduced key uncertainties concerning potential levels of internal exposure in humans. For example, we have learned that newborn and young rodents have significant age-dependent differences in metabolic capabilities, resulting in their not being able to metabolize BPA as well as adult rodents do and thus being exposed to higher levels internally; this is not the case for nonhuman primates (Doerge et al. 2010). Multiple pharmacokinetic studies in monkeys (supported by preliminary results in humans) have now demonstrated that newborn and young primates metabolize BPA at or very near the level of adult metabolism (Fisher et al. 2011; Patterson et al. 2013). Additional collaborative studies in pregnant primates have also shown both that potential fetal exposure is significantly reduced by the mother’s metabolic capabilities and that the fetus can effectively metabolize BPA (Doerge et al. 2010; Patterson et al. 2013).

Through the combined and collaborative efforts of the NIEHS and FDA, many important questions surrounding BPA and risks to human health have been or will be addressed in the near future. The promise of this collaborative approach between FDA regulatory researchers and NIEHS academic researchers extends well beyond BPA. It represents a new model for filling knowledge gaps and enhancing the value of investments in research, developing and promoting best methods and practices, informing chemical risk assessment, and identifying new methods or end points with the potential to improve regulatory hazard assessments and enhance protection of humans.

The goal of all our efforts is to support and perform the best science we can to inform the best possible decision making. Strong science is the common ground that can help us—particularly in situations of controversy where emotions and beliefs may become strong and positions polarized—to work together to accomplish what we all want to accomplish, keeping the public safe. Everyone involved in collaborative research has a role to play in building our understanding of the potential biological and/or health effects of substances and advancing technologies and methodologies, with the shared goal of protecting the public health.
